# Congenital Sepsis with *Candida albicans*—A Rare Event in the Neonatal Period: Report of Two Cases and Literature Review

**DOI:** 10.3390/microorganisms12091869

**Published:** 2024-09-10

**Authors:** Dumitru Alin Teacoe, Roxana Cristina Cormoș, Diana Adela Toma, Laura Ștef, Manuela Cucerea, Irina Muțiu, Radu Chicea, Dragoș Popescu, Eugen Dan Chicea, Adrian Gheorghe Boicean, Radu Galiș, Maria Livia Ognean

**Affiliations:** 1 Faculty of Medicine, Lucian Blaga University Sibiu, 550169 Sibiu, Romania; dumitrualin.teacoe@ulbsibiu.ro (D.A.T.); laura.stef@ulbsibiu.ro (L.Ș.); maria.ognean@ulbsibiu.ro (M.L.O.); 2 Clinical County Emergency Hospital Sibiu, 550245 Sibiu, Romania; 3 Department of Neonatology, George Emil Palade University of Medicine, Pharmacy, Science, and Technology, 540142 Targu Mures, Romania; 4 Municipal Hospital, 551026 Mediaș, Romania; 5 Department of Neonatology, Clinical County Emergency Hospital Bihor, 410167 Oradea, Romania; radu.galis@scjubh.ro; 6 Doctoral School, Poznan University of Medical Sciences, 60-535 Poznan, Poland

**Keywords:** congenital systemic candidiasis, candidemia, *Candida albicans*, congenital cutaneous candidiasis, invasive candidiasis, newborn, neonate, preterm infant, fluconazole

## Abstract

*Candida* spp. is rarely found in neonatal early-onset sepsis (EOS) etiology. However, candidemia is associated with increased mortality and morbidity, as in late-onset sepsis. Congenital candidiasis may present as a mucocutaneous infection or, more rarely, as a systemic infection in term and preterm infants. This paper presents case reports of two cases of congenital systemic candidiasis (CSC) caused by *Candida albicans* and a review of the data in the literature. An electronic search of PubMed, Scopus, and Google Scholar was performed to identify publications on congenital candidiasis. Both neonates were male, born vaginally, with risk factors for congenital candidiasis. One of the infants was born at term and presented with an almost generalized maculopapular rash at birth and congenital candidemia; parenteral fluconazole was used successfully. The other infant was born prematurely at 28 weeks of gestation; blood culture, gastric aspirate, and maternal vaginal cultures sampled at birth were positive for *C. albicans.* Liver and kidney involvement became apparent on the third day of life, while lung involvement was clinically evident on the fourth day. Prolonged parenteral fluconazole was administered due to multiple organ involvement and persistent candidemia. Our experience with the presented cases, similar to data in the literature, suggests that CSC may occur at any gestational age, with various clinical pictures, sometimes mimicking bacterial sepsis, and even in the absence of the rash. Careful anamnesis and a high index of suspicion are important for the prompt recognition and treatment of CSC, optimizing the short- and long-term outcomes. Further research should focus on CSC to improve its diagnosis.

## 1. Introduction

Neonatal sepsis is the third most common cause of neonatal death after prematurity and complications at birth [1]. Both early-onset sepsis (EOS) and late-onset sepsis (LOS) are associated with increased morbidity and mortality in neonates. The reported incidence of culture-proven neonatal EOS varies between 0.77–5/1000 live births [2,3,4,5,6]. Bacterial infections are the most common cause of neonatal EOS and LOS; however, despite their minor role in the etiology of neonatal sepsis, fungal infections can significantly impact neonatal morbidity and mortality.

*Candida* spp. are the leading cause of invasive fungal infections in infants admitted to neonatal intensive care units (NICUs). They are more commonly seen in LOS etiology than EOS (4.2 [7] to 9–26% [8] versus <1% [4] to 2.8% [9]). Neonatal EOS caused by *Candida* spp., also known as congenital candidiasis, is rarely seen. Its incidence is unknown but most probably underestimated [10], as the localized, cutaneous form is probably frequently mistaken for other neonatal dermatoses. A recent meta-analysis found less than 50 reports of generalized systemic candidiasis published in the literature in over 50 years [11] since the first report in 1958 [12]. *C. albicans* was isolated in blood, urine, and cerebrospinal fluid or identified by histological exams in various tissues or cultures sampled postmortem in most reported cases of congenital systemic candidiasis (CSC) [11,13]. The incidence of invasive *Candida* spp. infections was estimated at 5–10/100.000 live neonates [14,15,16]; an increased incidence of 5–15% is reported in very low birth weight infants (VLBW) as compared to 0.2–2% in the general population of infants admitted to the NICU [17]. Despite its rarity, CSC is a critical condition occurring in both term and preterm infants that is associated with increased morbidity and mortality if not recognized and treated promptly [18]. Conversely, increased awareness and prompt initiation of antifungal treatment in suspected cases, pending confirmation of the etiology, are crucial for improved outcomes.

We present two rare cases of congenital systemic candidiasis with *C. albicans.* The parents of the patients have given written consent for the publication of these case reports and associated images. We also received the approval of the Ethics, Medical Deontology, and Discipline of the Clinical County Emergency Hospital Sibiu, Romania, for publishing this report. Data in the literature regarding CSC was also reviewed.

## 2. Material and Methods

### 2.1. Case Reports

#### 2.1.1. Case Report 1

##### Medical History

**Figure 1:**
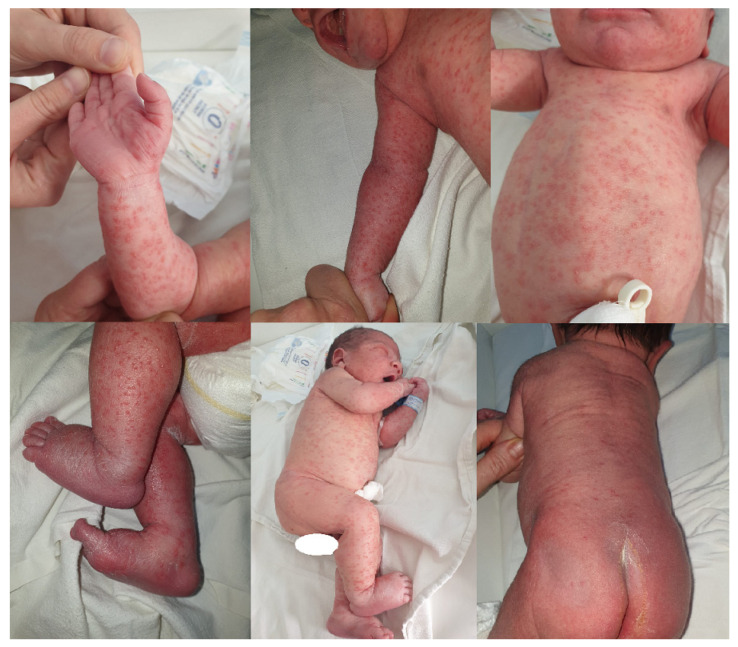
Cutaneous rash—aspects in the first 30 h of life.

The first case involves a full-term male newborn (39 gestational weeks) with a birth weight of 3600 g. The infant was delivered vaginally, in cranial presentation, and received an Apgar score of 10 at 1 min in a level I maternity hospital. The pregnancy progressed physiologically without any interventions or treatment. Approximately a week before delivery, the mother experienced leucorrhea and genital itching, untreated. Rupture of the amniotic membrane occurred during birth. At birth, the infant presented with a diffuse, generalized erythematous maculopapular rash and rare pustules, including on the palms and the soles; fewer lesions were noted on the posterior trunk and the extension surfaces of the legs; the buttocks, penis, scrotum, and perianal were spared (Figure 1); and no oral lesions were found. Staphylococcal maternal–fetal infection, abdominal tumor, and congenital heart defect were suspected, and the infant was transferred to our hospital after a few summary investigations.

##### Initial Assessment, Hematology, and Radiology Investigations

The infant was admitted to our NICU at 38 h of life. Upon admission, the newborn presented in good general condition: spontaneously breathing, respiratory rate of 46/minute, a peripheral oxygen saturation over 90% in room air, heart rate of 145 beats/minute, cardiac murmur grade II/6 on the left parasternal area, normal urine output, transitional stools, mild facial jaundice, good sucking reflex, normal tone and reflexes, and persistence of the above described cutaneous rash.

**Figure 2:**
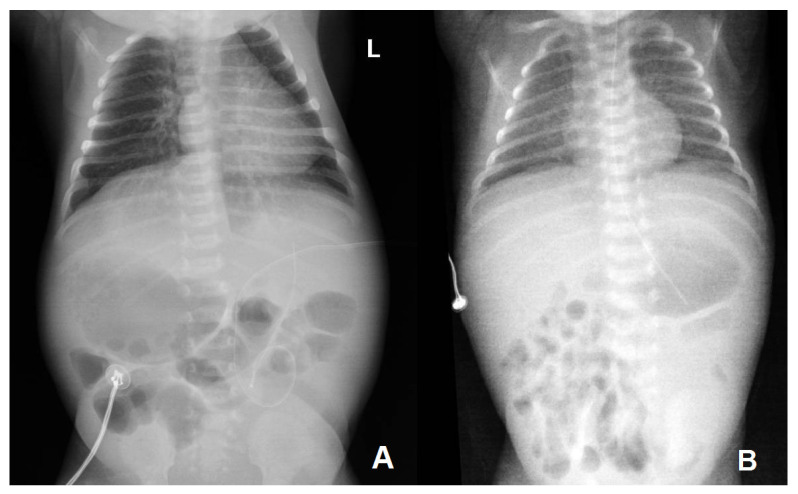
(**A**) Small bilateral perihilar interstitial foci; left-sided distended stomach, right-sided liver opacity, normal heart position in patient 1. (**B**) Slightly pronounced reticular, micronodular lung interstitium, bilateral.

Abdominal ultrasound revealed mirrored abdominal organs, suggesting incomplete abdominal situs inversus, confirmed by the thoracoabdominal radiography (Figure 2A). The head ultrasound was normal; the echocardiography revealed the presence of a small patent ductus arteriosus, patent foramen ovale, and a right-sided aortic arch. Intravenous administration of antibiotics (Penicillin G, 75,000 IU/kg/day, divided in 2 doses, and Amikacin, 15 mg/kg/day, once daily), according to the unit’s protocol in suspected EOS cases, was initiated for three days as C-reactive protein decreased rapidly to normal values (from 19.4 mg/L), and the infant showed no other signs or symptoms of infection.

##### Microbiology Investigations, Treatment

The maternal vaginal culture upon admission yielded negative results (Table 1). However, *C. albicans* grew in the blood culture sampled at admission, and yeasts were seen with a Gram stain on microscopy. Systemic fluconazole treatment (6 mg/kg/day) was initiated on the 4th day of life (DOL) and continued for 21 days, as the isolated strain of *C. albicans* showed sensitivity to this drug. Subsequent blood culture after 14 days yielded negative results. No other peripheral cultures were positive—nasal, pharyngeal swabs, gastric aspirate—except the cultured tip of the umbilical line (in situ for two days), which was positive for the same *Candida* strain (Table 2). The *Candida* isolate was sensitive to fluconazole (minimal inhibitory concentration (MIC), 0.5 mg/L), voriconazole (MIC 0.12 mg/L), amphotericine B (MIC 1 mg/L), flucytosine (MIC 2 mg/L), caspofungin (MIC 0.12 mg/L), and micafungin (MIC 0.06 mg/L).

##### Clinical Course and Outcome

Concurrently, the patient’s rash faded gradually by DOL 7, followed by fine, squamous desquamation, and ultimately resolved by 13th DOL (Table 1). The infant’s good general status, hemodynamic and respiratory stability, and efficient breastfeeding allowed the transfer, monitoring, and treatment of the infant in rooming-in from the 5th DOL. Repeated investigations during hospitalization did not reveal any involvement of other organ systems (Table 2). The infant was discharged after 24 days with instructions for scheduled follow-up. No significant health issues were observed up to 2 years of age, and the child’s growth and development were within normal range for his age.

#### 2.1.2. Case Report 2

##### Medical History

The second case involves to a premature male neonate born at 28 weeks gestation, weighing 1250 g, appropriate for gestational age. The infant was delivered vaginally at our level III maternity unit and received an Apgar score of 8 at 1 and 5 min. Immediately after stabilization, the preterm infant was admitted to the NICU. The pregnancy progressed uneventfully. The premature rupture of membranes (PROM) occurred 136 h before when the mother was admitted. Under tocolysis with hexoprenaline, a complete prenatal corticosteroid course (dexamethasone 4 doses of 6 mg, 12 h apart, intramuscularly) was administered as therapy for fetal lung maturation; additionally, the mother received intravenous ampicillin (2 g initially, followed by 1 g every 6 h) for five days and ceftriaxone (2 g, once, intravenous) for one day. The vaginal culture sampled from the mother at admission was negative. Another vaginal culture was sampled at birth (Table 1).

##### Initial Assessment, Investigations, and Management

Shortly after birth, the preterm infant presented signs of respiratory distress, including tachypnea (60 breaths/minute), grunting, intercostal retractions, and peripheral oxygen saturations of 75–80% in room air. Continuous positive airway pressure (Bubble CPAP) on the nasal cannula was used to support breathing. However, over the next 2 h, the oxygen need gradually increased up to 40%. At that point, non-invasive replacement therapy with surfactant was administered. According to the unit protocol, intravenous caffeine citrate, empiric antibiotic therapy (penicillin, 75,000 IU/kg/day, divided in 2 doses, and amikacin, 15 mg/kg/day, every 36 h), and parenteral nutrition were started. After surfactant administration, the oxygen need decreased to 21%, and normal blood and transcutaneous gases allowed complete weaning of the respiratory support after 72 h. An initial thoracic radiography showed a reticular pattern and air bronchogram consistent with respiratory distress syndrome due to surfactant deficiency. Since the initial values of the C-reactive protein were normal, the antibiotic therapy was stopped after 48 h.

##### Microbiology Investigations and Treatment

On the 4th DOL, the preterm infant experienced several episodes of desaturation, increased respiratory effort, and tachypnea. At 86 h of life, the C-reactive protein value was increased and the gastric aspirate culture collected at birth tested positive for *C. albicans*. When discussing the newborn’s condition with the mother, she mentioned that she was already receiving antifungal treatment (fluconazole) due to testing for *C. albicans* in the positive vaginal culture sampled at birth. As a result, antifungal therapy was started with intravenous fluconazole (12 mg/kg) along with intravenous antibiotic therapy with colistin (5 mg/kg/day, divided in 2 doses) and amikacin (15 mg/kg, every 36 h). The next day, the newborn’s blood culture collected at birth also showed positive results; the fungi gram showed sensitivity to fluconazole (MIC 0.5 mg/L), voriconazole (MIC 0.12 mg/L), amphotericin B (MIC 1 mg/L), flucytosine (MIC 1 mg/L), caspofungin (MIC 0.12 mg/L), and micafungin (MIC 0.06 mg/L). Due to increasing respiratory effort, prolonged desaturations, and a tendency to apnea, Bubble CPAP support was restarted. The chest radiography performed at this moment showed a slightly pronounced bilateral reticular, micronodular lung interstitium (Figure 2B). The respiratory support was reduced to humidified heated high-flow nasal cannula (HHHFNC) on DOL 15. A follow-up blood culture after 7 days of antifungal treatment showed again that candidemia. *C. albicans* was also found in the pharyngeal specimens and gastric aspirates sampled in the 4th DOL. Only the third blood culture, sampled 18 days after treatment initiation, was negative. At that time, the gastric aspirate, pharyngeal, and nasal swab cultures were also negative (Table 2).

##### Clinical Course, Complications, and Outcome

**Figure 3:**
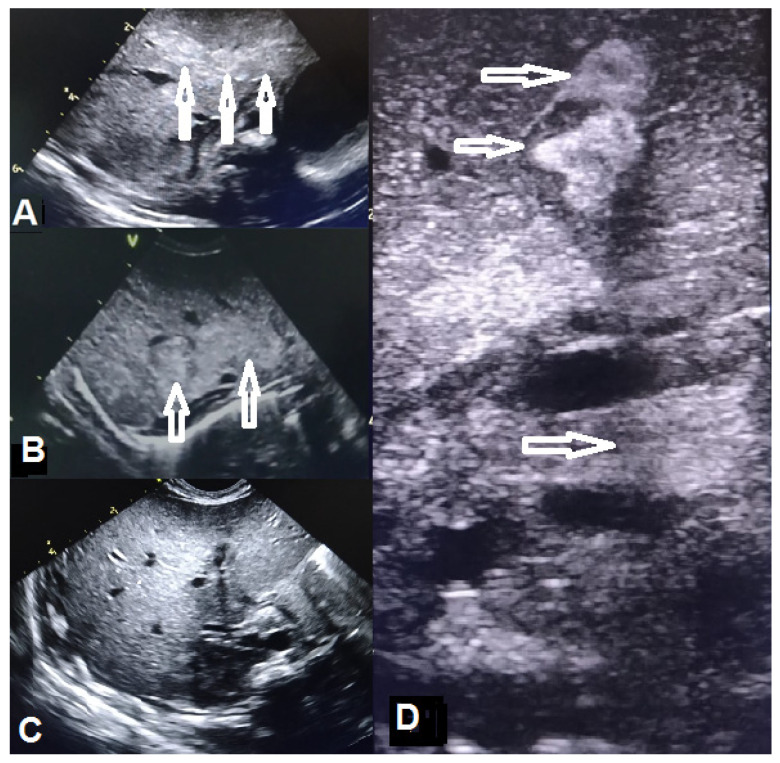
(**A**) Almost generalized gross, nodular, hyperechoic structure of the liver; (**B**,**D**) limited areas of fine granular echogenic hepatic structure; (**C**) fine, granular, normal sonographic aspect of the liver; arrows are indicating gross, nodular hyperechoic areas.

Abdominal ultrasound performed in the 9th DOL revealed hepatic involvement, characterized by generalized inflammatory changes—increased gross granular, hyperechoic patchy structure (Figure 3A), restricted after five days to the hepatic segment IV as a fine granular structure and altered echogenicity (Figure 3B,D), gradually fading to a normal smooth hypoechoic structure by the time of discharge. At that time, blood tests showed a transient mild increase in serum transaminases and renal markers (creatinine and urea), subsequently normalized by DOL 15. Fluconazole systemic therapy was maintained for 30 days. Head ultrasound was repeatedly normal for the age (Table 2).

The infant’s respiratory status gradually improved, and he was weaned off by DOL 41. In the context of prematurity, *C. albicans* sepsis, and respiratory support for more than 28 days, this was interpreted as mild bronchopulmonary dysplasia. The preterm infant was discharged recently, at 58 DOL, corrected postmenstrual age of 36 weeks, healthy, hemodynamically stable, oxygen-independent, partially breastfed, weight 3020 g, normal neurological exam, and recommendations for regular follow-up appointments up to the age of 2 years.

In both cases, a strict sterile technique and PedsPlus bottles were used to sample blood for culture before antibiotics were initiated; the bottles were incubated for 7 days using BD BACTEC^TM^ FX (Becton, Dickinson and Company, 7 Loveton Circle Sparks, MD, USA) blood system culture. Fungi were grown on Sabouraud Chloramphenicol Dextrose Agar (Condalab, LaboratoriesConda S.A., Madrid, Spian), and yeast cells and budding spores were identified microscopically on Gram stain.

### 2.2. Review of the Literature

**Figure 4:**
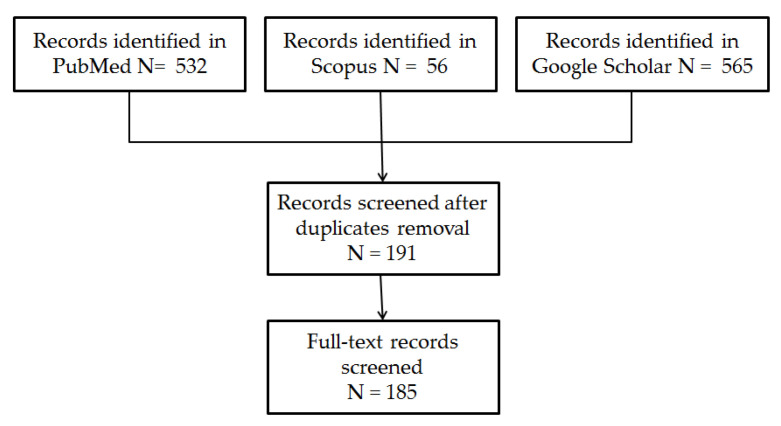
Flow diagram of the literature review.

Congenital candidiasis, whether cutaneous or systemic, is a rare disease. We aimed to review information on CSC published in the literature. Congenital invasive candidiasis was defined by *C. albicans* isolation in blood, urine, and cerebrospinal fluid in the first 7 DOL or identified by histological exams in various tissues or cultures sampled postmortem. PubMed, Scopus, and Google Scholar databases were searched using the following terms, in various combinations: “candidiasis”, “congenital”, “intrauterine”, “invasive”, “systemic”, “candidemia”, “Candida” and “newborn”, “neonate”, and “preterm infant”. We considered all types of publications, including case reports, case series, observational studies, clinical trials, reviews, systematic reviews, and meta-analyses, without any language restrictions. Additionally, we checked the reference lists of selected papers for further insights into congenital candidiasis congenital candidiasis. The review’s flow chart is presented in Figure 4. The selected papers were reviewed to gather updated information on congenital candidiasis. Updated information on CSC epidemiological and clinical aspects, risk factors, pathogeny, positive and differential diagnosis, treatment and prevention was revised and presented in the next chapter, parallel to the discussion of the presented cases.

## 3. Discussion

### 3.1. General Aspects

#### 3.1.1. Epidemiological Aspects

Neonatal sepsis is a critical condition associated with increased morbidity and mortality if not recognized and correctly treated in a timely manner. Although recently more frequently reported as an etiologic agent in neonatal sepsis, fungal infections are rarely identified in neonatal EOS [13,17,19,20,21,22,23,24,25,26]. Most of the reported cases occur as LOS, associated with prematurity, low birth weight, and NICU hospitalization [22,27,28,29]. *Candida* spp. are responsible for most neonatal fungal sepsis [13,18,19,22]. According to data in the literature, the incidence of neonatal EOS produced by *Candida* spp. varies between <1% [4] and 2.8% [9] compared to the incidence of 4.2% [7] to 9–26% [8] reported for neonatal LOS caused by *Candida* spp.

#### 3.1.2. Classification

Two types of congenital candidiasis are described: (a) localized, also called congenital cutaneous candidiasis (CCC), and (b) generalized, also referred to as congenital systemic candidiasis (CSC) (or invasive candidiasis). Generalized candidiasis is defined by positive blood or urine, or cerebrospinal fluid culture for *Candida* spp., or isolation of fungus in histopathological or cultures sampled at autopsy [11,13]. There is no consensus as regards defining early fungal sepsis; some authors consider sepsis within the first 72 h of life as early-onset [4,6,19,30], others use an extended timeframe of 7 days [26,31,32]. According to Gudjónsdottir et al. [6], it would not be appropriate to define neonatal sepsis as occurring after 72 h of life as early-onset sepsis (EOS) because the etiology changes significantly. For example, all cases with Group B Streptococcus, one of the main causes of EOS [4,6], are diagnosed within the first 72 h of life.

#### 3.1.3. Etiology

*C. albicans* is most commonly identified as a causal agent in congenital candidiasis [7,11,17,28,32,33,34,35,36], although cases attributed to *C. parapsilosis* [32,37,38], *C. glabrata* [39], *C. Kefyr* [40], and *C. tropicalis* [41] are also documented in the literature [13,31,42].

#### 3.1.4. Risk Factors

Multiple risk factors for congenital candidiasis have been reported, including prematurity, prolonged rupture of membranes, *Candida* vaginosis, and chorioamnionitis being the most frequent [11,13,23,29,34,35,36,40,42,43,44,45,46,47,48,49,50]. Notably, the risk of congenital candidiasis increases as gestational age and birth weight decrease [23,42,43]. A report from Canada [13] identifies an increased risk for congenital candidiasis in preterm infants < 25 weeks and birth weight < 750 g. Increased permeability of the immature epidermis, immature defense to fungal invasion, reduced opsonization and complement function, and immunoglobulin deficiency increase the risk for congenital candidiasis and invasive candidiasis [17,49,50,51,52]. On the other side, *Candida*’s adherence and slow growth facilitate its ability to colonize and disseminate in the blood and tissues even before clinical signs occur [17]. Even though most of the described cases of congenital candidiasis are associated with prolonged rupture of membranes, there are reports of neonatal *Candida* infection without amniotic membrane rupture prior to delivery [14]. *C. albicans* can penetrate the amniotic sac without evident membrane rupture [14,21,40,43,53], and the fetal infection can even develop without signs of vaginitis [54]. Additionally, many of the reported cases occurred in both vaginally and cesarean section-delivered infants [14,27,35,55,56].

*Candida* spp. are, in general, opportunistic, commensal fungi on the skin and reproductive and gastrointestinal tract. However, certain strains of *Candida* spp., such as *C. albicans* and *C. parapsilosis*, may produce candidiasis, increasing the risk of complications in pregnancy [44,46,47,53,57,58,59]. Up to 40–50% of the pregnant women are colonized with *Candida* spp. [13,44,60], with 13–20% of them presenting with *Candida* vaginitis [45,47,61,62], with *C. albicans* being responsible for 90% of the cases [46]. According to Disha et al. [46], three types of factors increase the risk of *Candida* vaginitis in pregnancy: (a) factors related to pregnancy—weakened immune system, high levels of estrogens and progesterone, low vaginal pH, decreased cellular-mediated immunity, increased glycogen content of the vaginal tissue; (b) clinical factors—diabetes, HIV infection, prior *Candida* infections; (c) behavioral factors—antibiotic or contraceptive use, intrauterine devices, wearing synthetic and tight clothes, deficient personal hygiene, inappropriate feeding, stress. In such circumstances, *Candida* may take advantage of the abnormal vaginal microbiome, microscopic breaches of the mucosa, or immune defense to invade the amniotic membrane and uterine cavity [11,21,47]. Despite the high incidence of fungal and *Candida* vaginitis, chorioamnionitis occurs rarely, and fetal and neonatal infections are even rarer [11,35,36,45,47,49], usually limited to the chorion and umbilical cord [11]. Intrauterine infection development is uncommon, with fewer than 100 reported cases of congenital candidiasis associated with *Candida* [53]. Nevertheless, the risk of congenital candidiasis increases in the rare event of systemic maternal fungal infection [40].

The presence of intrauterine devices [11,21,34,35,36,40,42,43,44,48,63], cerclage [21,48], and obstetrical procedures as assisted reproductive techniques [45], amniocentesis [11,21], embryo reduction [42], or chorionic villous sampling [47], as well as invasive maneuvers at birth [43], has been linked to an increased risk of congenital candidiasis of the offspring. Prior maternal antibiotic treatment, therapies producing an imbalance of the maternal vaginal microbiome (specifically destroying *Lactobacillus* spp.), glucocorticosteroids, oral contraceptives, hormonal substitution treatments, immunosuppressive conditions, maternal diabetes are also cited in the literature as being associated with congenital candidiasis [11,36,47,48,51,55,56,64,65]. Genetic predisposition has also been mentioned as a risk factor for *Candida* infection during pregnancy [36,51,55]. Additionally, an increased incidence of congenital candidiasis in male infants has been reported [66,67], possibly due to a gene on chromosome X involved either in thymus functioning or immunoglobulin synthesis, as proposed by Prinsloo et al. [68].

#### 3.1.5. Risk Factors for Invasive Congenital Candidiasis

Congenital candidiasis caused by *Candida* spp. is reported both in term and preterm infants, with a higher prevalence in preterm infants [11,48,49,67,69]. Despite the localized nature of CCC, caution is advised about its potential risk of dissemination, especially in preterm infants [14,21,34,70]. The risk is attributed to a more immature epidermal barrier [48,50] and innate and adaptative responses to pathogens [17,49,50,51]. The heightened risk for invasive and extensive neonatal and congenital candidiasis is also linked to low birth weight, the pathogen virulence, the magnitude of the inoculum, and invasive procedures [11,34,49,50,71].

#### 3.1.6. Comments on Reported Cases

In the past two years, we encountered two male infants with Candida-causing candidemia (CCC) in our unit. This represents an incidence of 0.31/1000 live newborns admitted in the maternity ward (2/6513 live births) and 2.89/1000 NICU admissions (2/691) between 2022–2024, which is higher compared to the generally reported data of 0.1% [17]. Both infants were delivered vaginally: patient 1 with amniotic membranes ruptured at birth, and patient 2 after 132 h of prolonged PROM. A history of vaginal discharge and itching was found in the mother of patient 1, although no fungi were detected in her vaginal cultures. The mother of the second patient had no history suggestive of vaginitis, and her vaginal culture sampled five days before birth was negative. However, a second vaginal culture sampled at birth was positive for *C. albicans*. A recent history of broad-spectrum antibiotic treatment and prophylactic dexamethasone for fetal lung maturation may have been associated with *Candida* transmission from the mother to the fetus in the second case. Research has shown that a longer duration of membrane rupture increases the risk of CSC [53], which was observed in the second patient, along with prematurity. Despite delayed clinical onset, associated inflammatory syndrome, and multiorgan involvement (lung, liver, and kidney) in the patient 2 (86 h of life), we classified the case as CSC, as the positive blood culture for Candida was sampled under sterile technique at 1 h of life, and the maternal vaginal swab obtained immediately after birth was also positive for Candida. The two isolated strains had similar sensibility to antifungal drugs on a fungal gram. Classifying neonatal sepsis as early- or late-onset based on onset timing is not yet universally accepted, as some experts define EOS as infections occurring up to the 7th DOL [26,31,72]. Of course, molecular typing of the isolated would have been of help for a clearer classification.

### 3.2. Physiopathology

#### 3.2.1. Transmission Pathways

Congenital infection with *C. albicans* is seldom acquired horizontally by the hematogenous spread of *Candida* spp. from the maternal circulation to the fetus through the placenta [11,17,21,33,36,40,42,45,47,51,73,74,75,76,77]. This pathway is universally associated with invasive visceral involvement, mainly affecting the liver [11,78], although any organ can be invaded—kidneys, spleen, brain—due to the immaturity of the neonatal immune responses [11,50]. A retrograde hematogenous seeding from the peritoneal cavity through the fallopian tubes has also been described [48].

More commonly, *C. albicans* reaches the fetus using the ascending route, penetrating the amniotic membranes into the amniotic fluid, resulting in inflammatory processes of the membranes, placenta, and umbilical cord [2,11,12,33,36,42,43,47,49,51,78,79]. The rupture of the amniotic membranes, presence of intrauterine devices or any foreign body in the uterus, and diagnostic or therapeutic obstetrical procedures are associated with the ascending route of transmission in congenital candidiasis [45,47,74,75,76]. Inhalation or ingestion of the infected amniotic fluid is proposed as a pathogenic mechanism for lung and gastrointestinal involvement in congenital candidiasis [11].

Following vertical transmission, *C. albicans* often colonizes neonatal skin and mucous membranes. Timely and adequate immune innate responses may prevent the excessive spreading of the fungus and subsequent infection of the newborn [80]. This may explain why congenital candidiasis usually presents as CCC in term infants, while preterm infants have an increased risk for invasive, disseminated disease, even if presenting initially as CCC [49,50].

#### 3.2.2. Comments on Reported Cases

The ascending transmission route seems more plausible in our patient 1; penetration of *C. albicans* through intact amniotic membranes, favored by maternal untreated vaginosis symptoms reported the week before delivery, has already been reported in association with congenital candidiasis [12]. In the second case, ascending access to chorion, placenta, amniotic fluid, umbilical cord, and the fetus was favored by the prolonged rupture of membranes and maternal treatment with broad-spectrum antibiotics and dexamethasone, all risk factors for fungal infection. Additionally, the maternal vaginal culture sampled at birth was positive for *C. albicans*. Nonetheless, the hepatic and renal involvement in patient 2 cannot exclude that *Candida* dissemination may have been favored by prematurity and umbilical line placement. The umbilical line placement was performed using a strict sterile technique; however, since *C. albicans* grew in the blood culture sampled from the umbilical line at its insertion, the line could have also contributed to the hematogenous spread of *C. albicans*.

### 3.3. Clinical Aspects

#### 3.3.1. Cutaneous Involvement

Recognition of congenital candidiasis is a challenge regardless of the clinical type, whether cutaneous or systemic. The cutaneous rash may present differently from one patient to another, and CSC presents with unspecific signs and symptoms, like most neonatal EOS [23,33,35,49].

Congenital cutaneous candidiasis rarely occurs [20,21,23,24]; its incidence is estimated at 0.1% of infants admitted to NICUs [14]. The cutaneous involvement is often apparent at birth or in the first days of life [14,43], no later than the sixth DOL [11,81]. In a series of CCC cases, Kaufman et al. [14] reported that the rash occurred in the first DOL in 71% of the patients at a median age of 0 days (0–6). The rash varies and may be diffuse erythematous, maculopapular, sometimes associating vesicles, bullae, or pustules, and is localized on the face, trunk, extension surfaces of the extremities, and intertriginous areas, often on the palms and soles [11,14,25,34,35,43,49,70,73,82,83,84]. The rash evolves in days with cutaneous desquamation, and different eruption stages can be seen simultaneously [23,70,73,83]; the genital area and oral mucosa are usually unaffected in CCC [82]. Keratin degradation, scaling, hyperkeratosis, parakeratosis, spongiosis, and vesiculation were demonstrated in an in vitro model of CCC in 72 h [85], another possible pathway for *C. albicans* dissemination. Rarely does CCC associate onychia and paronychia. Burn-like dermatitis has been reported, usually in preterm infants, and is associated with invasive fungal disease [45,82,86]. Mucocutaneous candidiasis is reported both in term and preterm infants; it most often has a benign, auto-limited course in term infants [11,43,82], resolving in 5 to 20 days [43], but is associated with risk of dissemination in preterm infants, especially the profound epidermal lesions, as burn-like dermatitis [70,82,87]. Recognition of CCC may be difficult as the rash may mimic various dermatologic conditions in the neonatal period [14,36,73]. Nevertheless, prompt recognition of CCC is important, as delayed treatment is associated with increased rates of dissemination and death [14,73].

#### 3.3.2. Systemic Involvement

Congenital systemic candidiasis is a rare condition compared to CCC [10], particularly in term infants [48], and is associated with a high mortality rate of 39–94% if left untreated [11,73,88,89]. Various clinical manifestations were reported in association with CSC, but the disease often presents with signs of respiratory distress due to lung involvement [11,20,28,37,70,82,90] and gastrointestinal manifestations [11,28,91]. Additionally, congenital candidemia may mimic bacterial EOS [33,49]. Pneumonia, cardiovascular, renal, liver, and ocular involvement have been reported in congenital candidiasis cases with *C. albicans* in extremely low birth weight infants in a study from Canada [13,33]. Furthermore, meningitis, brain abscesses, septic shock, intrauterine and perinatal death have also been reported in CSC [11,40,92,93]. The liver and spleen are affected in 1–3% of the cases of candidemia [8,49,94]. However, most of the cases diagnosed with hepatic abscess due to *Candida* spp. were identified in preterm infants with LOS with *Candida* spp. [95]. Early diagnosis of CSC poses a significant challenge due to its potential occurrence in the absence of identifiable risk factors, the unspecific clinical picture with a plethora of clinical signs, and absent rash in some cases [11,13,33,49,56]. Notably, the severity of CSC severity varies, from a subacute, indolent course to severe disease, with cardiorespiratory and multiple organ failure [49].

#### 3.3.3. Comments on Reported Cases

Based on data presented in the literature, the first of our patients, born at term, exhibited the typical rash in terms of onset (at birth), lesions (maculopapular, erythematous, rare pustule), extension and localization (almost generalized, with rare lesions on the buttocks, flexion face of the extremities, palms and soles, sparing the oral mucosa and genital and perianal areas), course (fading, in days, with furfuraceous desquamation), and resolution. No other involvement was found in this patient. Conversely, the preterm infant, patient 2, had no cutaneous eruption and experienced a relapse of respiratory distress on the 4th DOL. Investigations also revealed hepatic and renal involvement. Prematurity, respiratory distress syndrome due to surfactant deficiency, and normal initially C-reactive protein values had an important contribution in delaying CSC diagnosis.

### 3.4. Diagnosis

#### 3.4.1. Suspected Candidiasis at Birth

The first step in recognizing and diagnosing congenital candidiasis is a high index of suspicion, particularly in the presence of the risk factors [21,96], and in cases suspected of neonatal sepsis with an unsatisfactory clinical course despite an adequate therapeutic approach [90]. While most neonatal EOS cases are attributed to *Group B Streptococcus* and *Escherichia coli* infections, clinicians must be continuously aware that EOS may also be due to viruses, fungi, and parasites [4,6,19,49]. Hence, a meticulous examination of the placenta and the umbilical cord is mandatory when congenital candidiasis is suspected at birth. In cases of *Candida* spp. infection, yellow-white or red small nodules may be spotted on the umbilical cord (funisitis); on microscopic examination, these lesions are small subchorionic microabscesses with fungi and pseudohyphae [11,47,97]. Similarly, pseudohyphae, microabscesses, and/or granulomas can be found in the placenta [14,23,35,98]. Laboratory tests and imaging are helpful but have limited performance in diagnosing congenital candidiasis [25].

#### 3.4.2. Blood Culture

Blood culture is acknowledged as the primary method for diagnosing invasive candidiasis and CSC [11,13,17,23,29,33,34,49] due to its heightened specificity despite its limited sensitivity [33,99,100,101,102]. Blood cultures may also take time; with rapid tests, the results come in around 48–72 h [17,34,103], while classic methods may take 5–7 days for fungal growth [101,102,103]. A minimum of 1 mL of neonatal blood is required to minimize the rate of false negative results [8,33,101,102,103,104]. The failure to identify pathogens in the blood culture may be attributed to reduced pathogen inoculum, prior antibacterial and antifungal therapy, and viral or parasitic infections, and does not exclude infection in the presence of signs and symptoms suggestive of infection [104]. Experts also warn that a positive blood culture for *Candida* should not be casually dismissed as a contaminant, rather recognized as indicative of a potentially more profound systemic infection [102,105].

Modern technologies, such as real-time polymerase chain reaction (PCR), including multiplex PCR assays using a panel of *Candida* spp., may improve *Candida* spp. identification and offer a more rapid result [3,43,104,106,107]. However, studies are limited. Panfungal tests are not yet standardized, do not provide information on fungal sensitivity, and, despite high sensitivity and negative predictive value, their specificity and positive predictive value are unsatisfactory [33,34]. Additionally, a sensitivity of 90% and specificity of 93% were reported for molecular tests in diagnosing neonatal sepsis [108]. Nonetheless, studies report a high contamination rate and a potential risk of errors in interpreting the results [104]. Metagenomic sequencing allows simultaneous sequencing of billions of nucleic acid fragments at the same time and the rapid identification of bacteria, viruses, and fungi without distinction between pathogens and commensals. Data is limited in neonates, and these tests are time-consuming and expensive [33,109].

#### 3.4.3. Other Laboratory Investigations

Urine cultures can also help identify *Candida* spp., Cerebrospinal fluid cultures are indicated in the presence of signs of neurological infection [23,34,42,49]. A high suspicion of maternal *Candida* vaginitis or chorioamnionitis should prompt culture sampling from the maternal vagina, placenta, amniotic fluid, as well as sampling nasopharyngeal, gastric aspirates, and cutaneous lesions in the newborn [4,14,23,39,40,43,49]. Microscopic evaluation of the cutaneous lesions (e.g., Gram stain) may confirm fungal presence; however, the use of potassium hydroxide preparations should be avoided if scraping the skin is necessary for sampling [14]. A comprehensive laboratory evaluation is advised if blood culture is positive results for *Candida* to investigate the systemic involvement. Term newborns with mucocutaneous infection must undergo a thorough evaluation if there are concerns regarding other systemic infectious diseases [49].

The 1,3-β-D-glucan test (BDG) has been proposed as an alternative diagnostic tool in congenital candidiasis. BDG, a constituent part of the cellular membranes of multiple fungi, can be detected in the serum specifically in invasive fungal infections [33,110]. The cut-off values of BDG are not yet established in neonates [27,111]. The test can be used for treatment monitoring as it has an acceptable sensitivity (89%) but low specificity (60%) [33]. A better diagnostic accuracy may be achieved by combining BDG with clinical and imaging aspects and other laboratory data [17,110]. Serum mannan (an abundant constituent of the *Candida* cellular wall—acting as an antigen) or anti-mannan (antibodies) levels have minimal sensitivity, and the specificity of *Candida* spp. prevalence is low but may improve diagnostic accuracy in combination with blood culture [33].

Blood count and differential parameters have a limited value for congenital candidiasis diagnosis. Leukocytosis has been frequently reported in CSC [23,24,26,28,33,43,70,73,82,112]. Extreme leukemoid reactions associated with hyperglycemia and burn-like dermatitis were reported in three cases of CSC, all asymptomatic in the first 24 h of life by Pradeepkumar et al. [26]. Leukemoid reaction and increased number of immature neutrophils were found primarily on disseminated congenital candidiasis, associated with respiratory distress [23,113]. Although thrombocytopenia was associated with CSC [17,28,33,34], it cannot be considered a specific sign of *Candida* spp. sepsis [3,91,114]. Other authors also reported persistent hyperglycemia [23,26,33,40,70]. Recently, Ratridewi et al. [115] proposed a cut-off value of >5% monocytes as indicative of *Candida* spp. infection in preterm infants based on the important role played by monocytes in *Candida* invasion prevention.

Biochemical investigations can provide insights into the extension of *Candida* infection: aspartate aminotransferase (AST), alanine aminotransferase (ALT), alkaline phosphatase, bilirubin, triglycerides, gamma-glutamyl transferase (assessing liver function), creatinine, blood urea nitrogen (renal function), electrolytes, and inflammatory markers (most often use is C-reactive protein) [17,49].

#### 3.4.4. Imaging

Imaging examinations, including chest and abdomen X-rays, abdominal and head ultrasounds, and Doppler echocardiography, are helpful in the identification of fungal dissemination within lungs, liver, spleen, kidneys, cardiovascular system, and brain [17,40,43,49,94].

#### 3.4.5. Differential Diagnosis

Differential diagnosis of CCC and CSC rash (if present) encompasses various neonatal cutaneous eruptions, including toxic allergic erythema, milium, miliaria, transient neonatal pustular melanosis, or systemic conditions associated with rashes, such as neonatal EOS with *Listeria monocytogenes*, impetigo, cutaneous/systemic staphylococcal infections, congenital varicella, herpes virus congenital infection, epidermolysis bullosa, Langerhans cell histiocytosis, or toxic cutaneous reactions generated by drugs [11,14,23,24,63].

#### 3.4.6. Comments on Reported Cases

Laboratory tests, imaging, and the uneventful course suggested limited cutaneous infection with *C. albicans* in patient 1. All peripheral cultures sampled proved negative for *C. albicans*. No abnormalities were seen on his blood count and differential except an increased number of monocytes (12–12.5%) in the first two DOLs. Based on the maternal pregnancy and delivery history and tests, clinical aspects, imaging, and laboratory results, the patient was diagnosed with CSC with no other organ involvement except the skin. Patient 2, born prematurely, presented with two positive blood cultures for *C. albicans* (DOL 0 and 11), along with positive cultures from the gastric aspirate (DOL 0 and 4), pharynx (DOL 4), maternal vagina (day of birth), all indicating CSC. Persistent candidemia was mentioned in the literature in most newborns presenting with candidemia [8,116]. The delayed clinical onset of CSC (86 h of life) was suggestive of CSC dissemination of *C. albicans* acquired at birth, with lung involvement and associated with leukocytosis, monocytosis (DOL 1 and 4), hyperglycemia (DOL 4), increased AST (DOL 2–5), and creatinine (DOL 3–5). Additionally, the abdominal ultrasound performed on the 9th day revealed hepatic involvement.

### 3.5. Treatment

#### 3.5.1. General Aspects

Prompt initiation of antifungal therapy is associated with increased rates of survival in CSC [11,26,77]. However, there is a lack of consensus regarding the best management in CCC. Some experts advocate conservative treatment for all newborns presenting CCC and good general condition, reserving antifungal therapy for those with altered general condition [23,43,48,82] with concomitant evaluation for neonatal sepsis [82]. Continuous monitoring of well-appearing newborns with CCC without any sign of systemic infection and topical or oral antifungal therapy is advised by Salusti-Simpson et al. [48]. To reduce the risk of *Candida* dissemination, other sources suggest systemic antifungal treatment for more than 14 days initiated at the rash onset [14,25,42,73,96]. Systemic antifungal treatment is also recommended in infants with CCC associated with signs of respiratory distress, clinical or laboratory aspects suggestive of EOS, birth weight < 1500 g, broad-spectrum antibiotic therapy, extensive instrumentation at birth, weak immune system [11,23,43,48], in VLBW infants, and the presence of burn-like dermatitis lesions [14,23,48].

There is a stronger consensus supporting empiric systemic antifungal treatment when clinicians suspect *Candida* spp. infections in newborns at risk or with clinical signs of neonatal sepsis associated with recent thrombocytopenia or more than 50% reduction of the platelet count, gestational age < 26 weeks, or birth weight < 750 g [17,42,73].

#### 3.5.2. Antifungals

All authors agree that systemic antifungal therapy must be urgently administered if candidemia is present [11,17,18,26,27,33,42,43,73,77]. Three classes of antifungal drugs are available and have been used to treat neonatal candidiasis: (a) polyenes, (b) echinocandins (caspofungin, micafungin), and (c) azoles (fluconazole, itraconazole, voriconazole, posaconazole), each with various limitations in the treatment of invasive fungal infections [17,18]. Nephrotoxicity may limit polyenes usage; the limited antifungal spectrum and high cost limit echinocandins use [18]. Most authors recommend amphotericin B or fluconazole as the first-line antifungal treatment [11,17,27,28,33,35,42,45,48,73,117,118,119,120].

Amphotericin B, a polyene that produces pores in the fungal cell membrane due to interaction with ergosterol, may be used as amphotericin B deoxycholate (5 mg/kg) or liposomal amphotericin B (1 mg/kg) [33,43]. It is excreted in urine, but its use in urinary tract infections with *Candida* spp. is limited due to nephrotoxicity. Parenteral infusions are frequently associated with reactions and hypokalemia [18,33,121]. Amphotericin B can be used in *Candida* spp. infections resistant to fluconazole and to prevent resistance to fluconazole [122]. Additionally, 5-flucytosine has been recommended as a second-line therapy in *Candida* spp. in infections unresponsive to amphotericin B [117].

Fluconazole acts as a fungistatic by inhibiting the CYP450 enzyme and, consequently, ergosterol synthesis [33]. Fluconazole (6–12 mg/kg) exhibits an impressive safety profile and is efficient, particularly against *C. albicans* (some authors even recommend fluconazole as the first-line treatment for *C. albicans* candidemia [35]). It can be used parenterally, has an excellent enteral absorption of 90% [14,123], and has an accessible cost [17,18,124]. However, its use is limited by the increased risk of tolerance and association with persistent candidemia [125,126], increased resistance of *Candida* isolates, and recurrence risk [124,125]. Fluconazole has a limited efficiency against *C. glabrata* [45] and *C. parapsilosis* [28]. Both amphotericin B and fluconazole have good penetrance into the cerebrospinal fluid, being recommended in central nervous system fungal infections [33,35]. Caspofungin has been used in neonates with refractory and invasive fungal infections; however data in newborns is still limited [11,54,81].

#### 3.5.3. Supportive Treatment

Supportive treatment is mandatory, individualized according to the symptoms of each newborn with CCC: respiratory and cardiovascular support; antibiotic therapy, if necessary, must be tailored to pathogen resistance. Close monitoring of liver and renal function and electrolytes may require changes in fluid, nutrition, and electrolyte intake. Monitoring for signs of antifungal toxicity is also important. Repeated blood cultures and fungigrams may help identify *Candida* isolates resistant to the administered antifungal drug. For preterm infants, reducing or discontinuing humidification is advised if they are cared for in incubators with humidity [14]. Topical and oral antifungal therapy has been used and is recommended in cases evolving with CCC, usually nystatin or clotrimazole [1,43,82].

#### 3.5.4. Comments on Reported Cases

Parenteral fluconazole was immediately instituted once *C. albicans* infection was suspected and successfully used to treat CSC in both our patients without any side effects. The administration of a fluconazole course extended to 30 days due to multiple organ involvement (lung, liver, kidney), persistent candidemia after eight days of antifungal treatment, and a prolonged need for respiratory support and oxygen dependency. According to the study published by Benjamin et al. [127], 10% of the neonates, 320 infants in the study, exhibited multiple positive blood cultures and candidemia over 14 days.

### 3.6. Clinical Course and Complications

#### 3.6.1. General Aspects

The dissemination of CCC, more frequently observed in preterm infants, is associated with septicemia, meningitis, bronchopneumonia, arthritis, endocarditis, and increased mortality rates [11,14,26,31,54,77]. In 2010, Kaufman et al. [14] reported a dissemination rate of CCC of 66% in preterm infants weighing < 1000 g associated with a mortality rate of 40%. The dissemination rate was appreciated at 33% in preterm infants with a birth weight between 1000 and 2500 g, and their mortality rate was 14%. In infants weighing over 2500 g at birth, the dissemination rate dropped to 11% while the fatality rate decreased to 3.8%, underlying the need for systemic antifungal therapy for CCC in infants at risk for invasive fungal infections. Worse outcomes are cited for CSC with multiorgan involvement [21,24,43]. A meta-analysis on CSC published in 2020 reported mortality rates as high as 39 to 94%—higher in developing countries—associated with lower gestational age and birth weight and clinical onset at birth, especially with early-onset respiratory distress [11]. While outcomes may vary according to the level of intensive care, mortality in *C. albicans* infections is increased compared to *C. parapsilosis* and other non-*C. albicans* sepsis (approximately 40% versus 15%) [17].

Similar to bacterial neonatal EOS, CSC may be associated with increased hospitalization costs and poorer developmental outcomes, especially in preterm infants, including an increased risk for cerebral palsy, blindness, and deafness [17,33,128].

#### 3.6.2. Comments on Reported Cases

Both our patients had a favorable short-term outcome. The first patient was monitored for two years, had no significant health problems, and demonstrated appropriate-for-age growth and development at the age of 2 years. Prematurity, multiorgan involvement, and persistent candidemia may have contributed to bronchopulmonary dysplasia in patient 2; long-term follow-up was scheduled for this infant, too. Encouragingly, the good clinical status and growth and resolution of all organ involvement up to discharge at 36 weeks corrected age is encouraging for a favorable long-term prognosis.

### 3.7. Prevention

Recognizing the significant impact of maternal *Candida* spp. colonization and vaginitis, as well as the profound implications of congenital candidiasis on neonatal morbidity and mortality, some have established screening programs for prenatal fungi detection [36]. Furthermore, maternal prenatal treatment in the last trimester of pregnancy, even in asymptomatic colonization, has substantially reduced *Candida* transmission from the mother to the offspring and oral and genital candidosis in neonates [129,130].

## 4. Study Limitations

We acknowledge the limitations inherent in our study, as it is based on the report of two cases. Nonetheless, we extensively reviewed the literature on congenital candidiasis, with particular focus on CSC. In presenting our cases, we emphasized the practical management of the patients. All the data for the first patient is presented in this paper. As for the preterm infant, a very low birth weight infant, we have chosen to present only the clinical and paraclinical information relevant for the first month of life, as the rest of the hospitalization period was uneventful, and the prolonged hospital stay was due to prematurity and bronchopulmonary dysplasia. In both cases, we followed the unit protocol for suspected neonatal sepsis and used our laboratory resources. According to our protocol, lumbar puncture is performed only if clinical signs and symptoms or a head ultrasound suggest central nervous system involvement. If the blood culture is positive, serial head ultrasounds are performed at least every 3–5 days, more often if needed, at the clinician’s discretion. Additionally, our protocols limit blood sampling for tests in well-appearing infants unless a condition is highly suspected. In aligning with best practices, we sought to adopt the most appropriate management. In this pursuit, we recognized the rarity of this condition, with only 43 cases found in the meta-analysis published four years ago and very few additional cases since then [28,56,73], totaling less than 50 cases since 1958 when the first case of CSC [12] was published.

## 5. Conclusions

We reported two cases of *C. albicans* CSC, both with distinct characteristics. A full-term infant was diagnosed with CSC, with a positive blood culture sampled in the first DOL. The diagnosis was prompted by the presence of the characteristic rash at birth, suspected neuroblastoma, and congenital heart defect. Treatment with fluconazole was successful and led to a favorable outcome, and both the short- and long-term outcome was favorable.

In the case of the preterm infant, CSC diagnosis was slightly delayed by the infant’s presentation at birth with respiratory distress syndrome, which was attributed to surfactant deficiency due to prematurity. This was quickly resolved by surfactant therapy, non-invasive respiratory support, and oxygen therapy. The infant’s C-reactive protein values were normal in the first two days of life. The maternal vaginal culture initially tested negative, and the infant’s blood culture results, sampled at birth, were delayed. Most probably, all these circumstances have favored *C. albicans*’ spread to the liver and kidney, with minimal clinical impact on the infant’s condition. However, lung tissue infection most probably contributed to the development of bronchopulmonary dysplasia. A longer systemic fluconazole therapy was deemed necessary due to the persistent presence of *C. albicans* in the blood. A favorable short-term outcome was achieved as the infant was discharged after an uneventful course, growing and developing within normal limits at 36 weeks of corrected age. Notably, both infants presented monocytosis at the clinical onset of CSC. Machine learning or artificial intelligence could aid in evaluating the integration of the monocyte count into a diagnostic algorithm for congenital candidiasis.

The literature review and our recent experience with the reported cases have provided insights that align with those expressed by other authorities in the field: (1) A high index of suspicion in the presence of risk factors for congenital candidiasis is important for early recognition, prompt and appropriate antifungal treatment, and outcome optimization. (2) CCC are rare events and CSC even rarer. Even if, in well-appearing term infants, CCC has a benign, auto-limited course, there are multiple circumstances in which CCC can disseminate, leading to invasive fungal infection with multi-organ involvement and high mortality rate. Such cases must be considered for systemic antifungal treatment. (3) Variable and unspecific clinical aspects challenge the clinical recognition of congenital candidiasis. (4) Routine tests utilized for neonatal candidiasis are beset with numerous limitations. A negative blood culture—the gold standard test for neonatal sepsis—does not exclude candidemia. Rapid, newer tests and technologies may help rapid diagnosis. In the presence of prenatal risk factors, placenta, amniotic membranes, and umbilical cord examination may offer a clue for diagnosis. (5) Both amphotericine B and fluconazole are effective in CCC and CSC treatment; however, long-term therapy is often needed, increasing hospitalization costs. It is evident that a standardized approach is needed to diagnose and manage congenital candidiasis, a rare disease posing an increased burden of morbidity and mortality on patients and their families, clinicians, and healthcare systems [13,14,23,24,42,73,89]. Particular emphasis should be placed on the recognition and treatment of congenital candidiasis, particularly CSC, given its elevated complexity and heightened risk of adverse clinical outcomes.

## Figures and Tables

**Table 1:** Maternal history, clinical characteristics, and clinical course of the two patients.

	Patient 1	Patient 2
Gestational age	39 weeks	28 weeks
Birth weight	3600 g	1250 g
Maternal history	Vaginal discharge and genital itching one week before delivery	132 h of membrane rupture, intravenous ampicillin for 5 days, associated with ceftriaxone at birth
Maternal vaginal cultures	No pathogen growth at admission in our unit (2nd day after delivery)	No pathogen growth 5 days before delivery, *C. albicans* was isolated in cultures sampled at birth
Clinical onset	At birth, with characteristic rash	The 4th day, respiratory distress syndrome
Clinical course	No other signs or symptoms suggestive of sepsis, rash entirely resolved by DOL 13	Re-initiated respiratory support (after initial treatment for respiratory distress syndrome due to surfactant deficiency with Bubble CPAP and surfactant), weaned on HHHFNC on DOL 15, oxygen-independent at DOL 41
Complications	None	Hepatic and renal involvement
Discharge	DOL 24	DOL 58
Follow up	Normal growth and development at the age of 2	Recently discharged, follow-up scheduled

Legend: DOL—day of life, CPAP—continuous positive airway pressure, HHHFNC—humidified heated high-flow nasal cannula.

**Table 2:** Relevant laboratory and imaging investigations of the two patients.

	Patient 1					Patient 2							
	12 h	Day 1	Day 3	Day 12	Day 17	12 h	Day 1	Day 2	86 h	Day 5	Day 10	Day 15	Day 20
Blood count and differential													
Platelets (10^3^/µL) (range 150,000–400,000) ‡	434	412	389	354	-	-	374	-	-	331	356	-	-
Leucocytes (/µL) (range 9000–26,000) ‡	17,440	14,430	11,280	9760	-	-	23,300	-	-	21,240	15,050	-	-
Neutrophils (/µL) (range 2700–14,400) ‡	10,580	7540	6820	5730	-	-	15,090	-	-	14,350	9030	-	-
Monocytes (%) (range 0–2%) ‡	1.4	12	12.5	4.1	-	-	8.4	-	-	9.5	5	-	-
Inflammatory markers													
C-RP (mg/L) (normal value < 5 mg/L)	19.38	8.3	7.4	1.6	3.1	2.1	1.3	<1	41.4	36.6	14.3	12.5	2.9
Biochemistry													
Blood glucose (mg/dL) (range 40–60 mg/dL 0–24 h, 40–100 mg/dL > 24 h of life)	63.1	74	85	-	-	59	77	57	123	49	87	89	78
AST (U/L) (range 11–70 U/L)	38.4	34	29	27	15	29	29	114	107	87	20	27	28
ALT (U/L) (range 0–70 U/L	13.3	16	18	15	14	<7	<7	14	21	22	9	8	9
Creatinine (mg/dL) (range 0.31–0.53 mg/mL)	1.01	0.61	0.52	0.54	0.45	0.52	0.85	0.97	1.15	1.25	0.91	0.75	0.54
BUN (mg/dL) (range 11–36 mg/mL)	30.5	12	13	15	24	24	63	83	79	74	37	26	19
Total bilirubin (mg/dL) ^§^	3.4	4.3	5.5	1.2	0.17	2.3	4.6	6.8	6.9	6.9	1.7	-	-
Imaging													
Thoraco-abdominal radiography	No lung or abdominal involvement				Suggestive of respiratory distress syndrome due to surfactant deficiency on DOL 0; pronounced reticular, micronodular bilateral lung interstitium on DOL 5								
Abdominal ultrasound	Abdominal situs inversus on DOL 2; no abdominal parenchymal involvement on DOL 2 and 10				Gross, inhomogenous, patchy echogenic areas disseminated, almost throughout the entire liver on DOL 9; fine granular echogenic areas limited to the fourth hepatic segment by DOL^6^ 14; normal hepatic ultrasound structure on DOL 45								
Doppler echocardiography	Small PDA, PFO, right-sided aortic arch (DOL 1)				Small PDA, PFO on DOL 4 and 14								
Microbiology													
Blood culture	Positive for *C. albicans* at admission, no growth at 14 days				Positive for *C. albicans* on DOL 0 and 11, negative on DOL 18								
Nasal swab culture	No pathogen identified				No pathogen identified on DOL 4 and 18								
Gastric aspirate	No pathogen identified				*C. albicans* isolated on DOL 0, and 4, no pathogen growth on DOL 18								
Pharyngeal culture	No pathogen identified				*C. albicans* isolated on DOL 4, no pathogen growth on DOL 18								
Umbilical line tip culture	*C. albicans* (in situ for 2 days)				*C. albicans* (in situ for 5 days)								

Legend: C-RP—C-reactive protein; AST—aspartate aminotransferase; ALT—alanine aminotransferase; BUN—blood urea nitrogen; DOL—day of life; PDA—persistent ductus arteriosus; PFO—persistent foramen ovale; **^§^**—serum bilirubin values are to be interpreted according to the gestational age, chronological age, and risk factors for hyperbilirubinemia; ‡—normal values during the first week of life.

## Data Availability

All relevant data regarding the cases presented in the study are included in the article; further inquiries can be directed to the corresponding author. Restrictions may apply due to institutional policies.
